# Height of Rectal Cancer: A Comparison between Rectoscopic and Different MRI Measurements

**DOI:** 10.1155/2020/2130705

**Published:** 2020-01-28

**Authors:** U. I. Attenberger, J. Winter, F. N. Harder, I. Burkholder, D. Dinter, S. Kaltschmidt, P. Kienle, S. O. Schoenberg, R. Hofheinz

**Affiliations:** ^1^Department of Clinical Radiology and Nuclear Medicine, University Hospital Mannheim, Medical Faculty Mannheim, Heidelberg University, Mannheim, Germany; ^2^Department of Nuclear Medicine, Heidelberg University Hospital, Heidelberg, Germany; ^3^Department of Diagnostic and Interventional Radiology, University Hospital Rechts der Isar, Technical University Munich, Munich, Germany; ^4^Department of Nursing and Health, University of Applied Sciences of the Saarland, Saarbruecken, Germany; ^5^Radiologie Schwetzingen, Schwetzingen, Germany; ^6^Radiologie Westpfalz-Klinikum, Kaiserslautern, Germany; ^7^General and Visceral Surgery, Theresienkrankenhaus and St. Hedwig-Klinik GmbH, Mannheim, Germany; ^8^Department of Medical Oncology, University Hospital Mannheim, Medical Faculty Mannheim, Heidelberg University, Mannheim, Germany

## Abstract

**Purpose:**

To compare rigid rectoscopy with three different MRI measurement techniques for rectal cancer height determination, all starting at the anal verge, in order to evaluate whether MRI measurements starting from the anal verge could be an alternative to rigid rectoscopy. Moreover, potential cut-off values for MRI in categorizing tumor height measurements were evaluated.

**Methods:**

In this retrospective study, 106 patients (75 men, 31 female, mean age 64 ± 11.59 years) with primary rectal cancer underwent rigid rectoscopy as well as MR imaging. Three different measurements (MRI1–3) in T2w sagittal scans were used to evaluate the exact distance from the anal verge (AV) to the distal ending of the tumor (MRI1: two unbowed lines, AV to the upper ending of the anal canal and upper ending of the anal canal to the lower border of the tumor; MRI2: one straight line from the AV to the lower boarder of the tumor; MRI3: a curved line beginning at the AV and following the course of the rectum wall ending at the lower border of the tumor). Furthermore, agreement between the gold standard rigid rectoscopy (UICC classification: low part, 0-6 cm; mid part, 6-12 cm; and high part, >12 cm) and each MRI measuring technique was analyzed.

**Results:**

Only a fair correlation in terms of individual measures between rectoscopy and all 3 MRI measurement techniques was shown. The proposed new cut-off values utilizing ROC analysis for the three different MRI beginning at the anal verge were low 0-7.7 cm, mid 7.7-13.3 cm, and high > 13.3 cm (MRI1); low 0-7.4 cm, mid 7.4-11.2 cm, and high > 11.2 cm (MRI2); and low 0-7.1 cm, mid 7.1-13.7 cm, and high > 13.7 cm (MRI3). For MRI1 and MRI3, the agreement to the gold standard was substantial (*r* = 0.66, *r* = 0.67, respectively).

**Conclusion:**

This study illustrates that MRI1 and MRI3 measures can be interchangeably used as a valid method to determine tumor height compared to the gold standard rigid rectoscopy.

## 1. Introduction

Rectal cancer is a leading cause of cancer death worldwide [1, 2]. In order to plan treatment, knowledge of tumor localization, distance of the tumor to the circumferential resection margin (CRM), tumor extension (T stage), extra mural venous invasion, and lymph node metastases are fundamental [3]. The primary tumor is usually identified by digital palpation and endoscopy with biopsy. For local staging, magnetic resonance imaging (MRI) has achieved broad acceptance prior to treatment due to its high diagnostic accuracy in the assessment of the mesorectal fascia [4]. Endorectal ultrasound is only recommended by the current guidelines to assess T1 tumors [3].

Besides the infiltration of the CRM, tumor height is an essential factor in the assessment and treatment stratification of rectal cancer. Tumor height is generally defined as the distance from the anal verge to the lower boarder of the tumor. Alternative measurement defines tumor height from the anorectal junction to the lower board of the tumor.

An accurate determination of the tumor height is crucial because T3 tumors located in the upper third are generally not treated with neoadjuvant chemoradiation. While, proximal rectal cancer spreads along the superior vessels, lower rectal cancer metastasizes along the lymphatic route of the mid rectal to internal iliac vessels. The route downward along the inferior rectal vessels is rare and only seen in very low rectal cancer, mostly accompanied by an infiltration into the anal canal [5]. Another important factor impacting further treatment strategies is the distance of the tumor to the mesorectal fascia (MRF). Tumor infiltration into the mesorectal fascia leads to a higher local recurrence rate [5, 6]. Furthermore, tumor localization within the peritoneal reflection is an important risk factor, since tumor infiltration into the peritoneal reflection is defined as stage T4a.

Measured by rigid endoscopy, the UICC classifies tumors beginning at the anal verge up to 6 cm as low rectal cancer, between 6 and 12 cm as mid rectal cancer, and between 12 and 16 cm as high rectal cancer [7]. The European Society for Medical Oncology (ESMO) sets the cut-off values for rigid rectoscopy by <5 cm beginning at the anal verge as low, 5-10 cm as mid, and 10-15 cm as high rectal cancer [3]. Since the standard rigid rectoscopy measurement is taken from the anal verge [8], the anal canal is included in the measurement.

For MRI measurements, ESMO sets the cut-off beginning at the anorectal junction up to 4 cm for low, from 4 to 8 cm for mid, and from 8 up to 12 cm for high rectal cancer [3]. The anal verge is the defined standard reference line for tumor height measurements in rectoscopy. Since its identification is more simplistic than identifying the anorectal junction in MRI, the anal verge might also be a superior landmark for MRI measurements than the anorectal junction. Several studies have shown that the length of the anal canal depends on the patient's sex, length, and weight [9]. For men, the surgical anal canal was 4.4 (3.2-5.3) cm and for women 4.9 (3.0-5.0) cm [10]. Which measurement technique (straight vs. curved line along the rectum) might be most accurate needs to be evaluated. MR measurement is proposed by the ESMO for the determination of rectal cancer height; however, so far, the appropriateness for clinical use has never been further evaluated.

Today, the most common modality for assessing the tumor location is the rigid rectoscopy. All major trials conducted to date have no defined common starting point for tumor height measurement by MRI [8]. Although the complication rate is low, rigid rectoscopy is an uncomfortable examination for the patient. MRI in contrast is a noninvasive technique, and furthermore, the recommended modality of choice for initial local staging prior to treatment.

Thus, the aim of this study was to compare rigid rectoscopy with different MRI measurement techniques (straight vs. curved) all starting at the anal verge in order to evaluate whether MRI measurements starting from the anal verge could be an alternative to rigid rectoscopy. Moreover, potential cut-off values for MRI in categorizing tumor height measurements were evaluated.

## 2. Material and Methods

### 2.1. Patients

106 patients (75 men, 31 female, with a mean age of 64 ± 11.59 years) were enrolled in this IRB-approved, retrospective study. After written informed consent, all patients underwent a rigid endoscopy as well as a MRI at 3 and 1.5 T between 5/2009 and 11/2012 as part of an institutional standard workup for local staging of locally advanced primary rectal cancer.

### 2.2. Imaging Protocol

Except for two MRIs which were performed at a 1.5-tesla MR system (Avanto, Siemens Healthineers, Erlangen, Germany), the remainder were performed at a 3-tesla MR system (MAGNETOM TimTrio, Siemens Healthineers, Erlangen, Germany) utilizing a standard 6-element body-matrix coil centered over the pelvis in combination with the built-in 32-element spine-matrix coil. 50 mL of ultrasound gel was inserted into the rectal vault of each patient prior to the initial MRI procedure. Our standard rectal cancer protocol as previously published elsewhere [11] has been used, consisting of standard T2w TSE scans, acquired in axial, coronal, and sagittal planes for anatomic orientation after localizer sequences. The axial and coronal T2w sequences were angulated perpendicular and parallel to the tumor axis. A high-resolution T2w TSE axial scan (TR/TE/FA 4000 ms/101 ms/150°, FoV 200 × 200 mm^2^, matrix 320 × 310), parallel imaging (GRAPPA) factor 2, and DWI (TR/TE/FA 5000 ms/73 ms/90, FoV 284 × 379 mm^2^, matrix 115 × 192, *b* = 50/400/800/1000 s/mm^2^), parallel imaging (GRAPPA) factor 2, were acquired. An additional echo-shared, high spatial and temporal resolution, and time-resolved MR examination with interleaved stochastic trajectories (TWIST-MR) (TR/TE/FA 3.6/1.4/15 ms/ms/°, FoV 350 × 187 mm^2^, matrix size 192 × 144, slice thickness 3.6 mm, temporal resolution 4.9 s) in an axial oblique slice orientation was acquired for the perfusion analysis. A Gd-based 0.5 M macrocyclic contrast agent (Dotarem, Guerbet, France) was injected at a dose of 0.1 mmol/kg/body weight and at a rate of 2.5 ml/s, followed by 40 ml of saline flush, injected at the same rate.

### 2.3. Image Analysis

**Figure 1:**
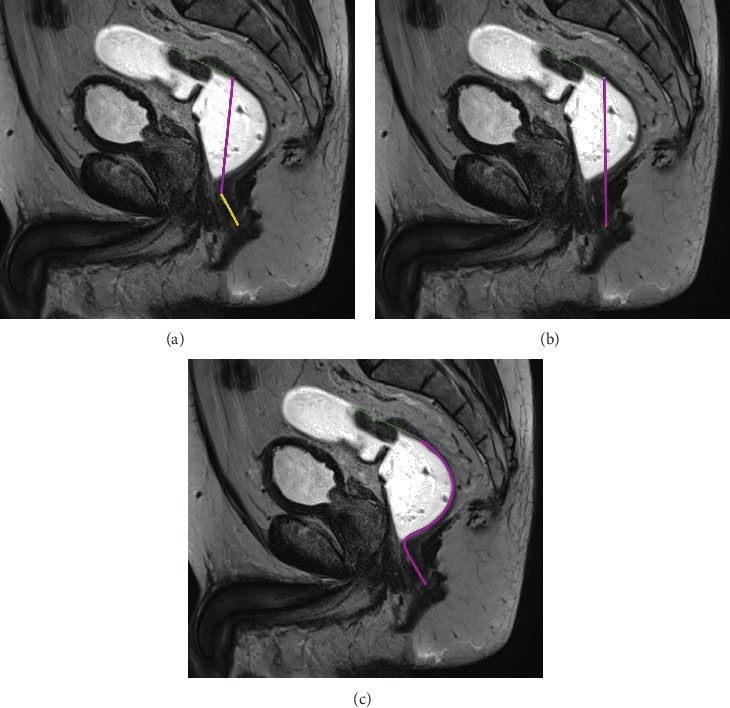
(a) The figure shows two unbowed lines, one from the anal verge to the upper ending of the anal canal (yellow) and the other beginning at the upper ending of the anal canal to the lower border of the tumor (purple) on a high-resolution T2w sagittal scan. The green line indicates the length of the tumor. (b) The figure shows one straight line (in purple) from the anal verge to the lower border of the tumor on a high-resolution T2w sagittal scan. The green line indicates the length of the tumor. (c) The figure shows a curved line beginning at the anal verge and following course of the rectum wall ending at the lower border of the tumor (purple) on a high-resolution T2w sagittal scan. The green line indicates the length of the tumor.

Images were evaluated on an OsiriX Workstation (Version 5.6, Pixmeo Inc., Geneva, Switzerland) by two independent radiologists. Three different measurements (BDM) in a single 2D image were used to evaluate the exact distance from the anal verge to the distal ending of the tumor. All geometric measurements were performed on the T2w sagittal scans. Diffusion-weighted images, DCE scans, and the high-resolution T2w axial scan were used for improved tumor identification. For the first measurement (MRI1), two unbowed lines, one from the anal verge (AV) to the upper ending of the anal canal and the other beginning at the upper ending of the anal canal to the lower border of the tumor, were drawn and both distances were added (Figure 1(a)). In the second measurement (MRI2), one straight line from the anal verge to the lower boarder of the tumor was measured (Figure 1(b)). The third measurement (MRI3) was a curved line beginning at the anal verge and following the course of the rectum wall ending at the lower border of the tumor (Figure 1(c)).

### 2.4. Rigid Rectoscopy

Rigid rectoscopy and endoscopic ultrasound were performed by experienced endoscopic surgeons. Cut-off values were set in the following way: low (0-6 cm), mid (6-12 cm), and high (>12 cm). The anal verge was used as the baseline reference level.

### 2.5. Statistical Analysis

Bland-Altman plots were used to compare agreement between the four different measurement techniques (rigid rectoscopy and three different MRI measurement techniques). Normal distribution was analyzed by using the Shapiro-Wilk test. In case of nonnormality, the log-transformed values were used for analysis and limits of agreement were back transformed to the original scale. The Friedman test was applied for analyzing overall differences between measurement techniques. If significant overall differences were found, post hoc analysis using a paired Wilcoxon signed-rank test was performed comparing all methods with each other in groups of two applying the Holm-Bonferroni method to adjust *p* values of paired tests regarding multiple comparisons.

In addition, a ROC (receiver operating characteristic) analysis was performed separately for each MRI measuring technique. The sensitivity and the specificity were calculated first for each value observed, and the ROC curve was generated. In addition, the Youden index (Youden index = Sensitivity + Specificity − 1) was calculated for each value. As the optimal cut point for the lower part, the maximum Youden index was determined. The optimal cut point for upper part was found by maximizing the weighted kappa coefficient using the optimal cut point for the lower part.

Statistical analysis was performed using the SAS statistical package for Windows version 9.3 (SAS Institute Inc., North Carolina, USA). Bland-Altman plots were performed using R Version 2.15.2.

## 3. Results

### 3.1. Mean Values

**Figure 2:**
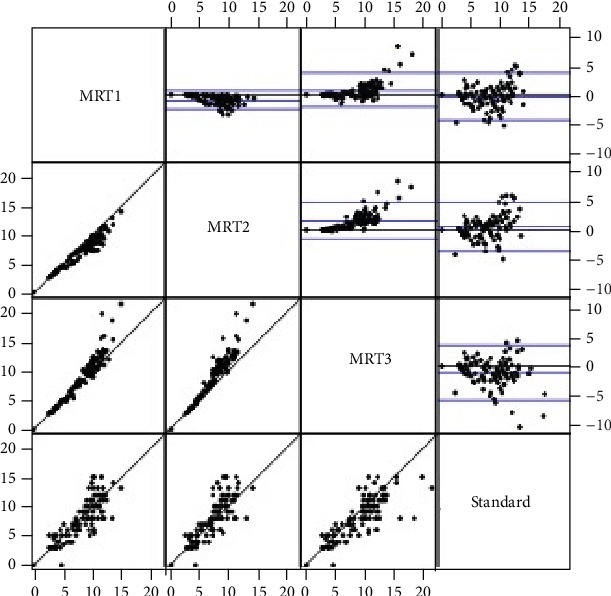
Bland-Altman analysis.

Bland-Altman analysis with back transformation to the original scale revealed that values for MRI1 were in mean 6% (mean ratio: 0.94, 95% limits of agreement (0.58, 1.53)) and MRI3 values in mean 11% (mean ratio: 0.89, 95% limits of agreement (0.54, 1.49)) above the rectoscopic measurements. Values for MRI2 were in mean 5% below the rectoscopic measurements (mean ratio: 1.05, 95% limits of agreement (0.64, 1.70)).  Figure 2 displays the Bland-Altman analysis.

**Figure 3:**
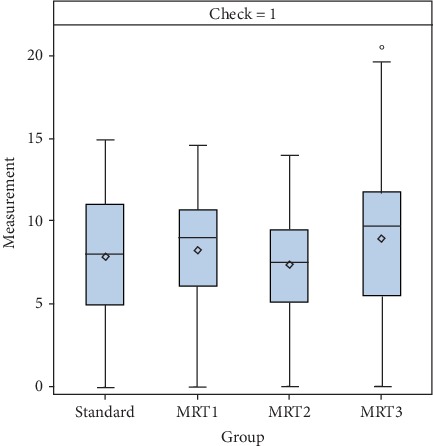
Mean values ± standard deviations for the four different measurement techniques of the height of the rectal cancer.

Figure 3 and Table 1 illustrate the mean values ± standard deviations for the four different measurement techniques (rigid rectoscopy, MRI1, MRI2, and MRI3).

Comparing the three different MRI measurement techniques (MRI1–3) to the results of the rectoscopic measurements, statistically significant difference was found in all instances except for MRI1: rectoscopy-MRI1: adjusted *p* = 0.05; rectoscopy-MRI2: adjusted *p* = 0.028; and rectoscopy-MRI3: adjusted *p* = 0.006. Comparing the MRI measurements, all results were significantly different among the different measurement techniques: MRI1–MRI2: adjusted *p* < 0.006; MRI1–MRI3: adjusted *p* < 0.006; MRI2–MRI3: adjusted *p* < 0.006.

#### 3.1.1. ROC-Curve Analysis

**Figure 4:**
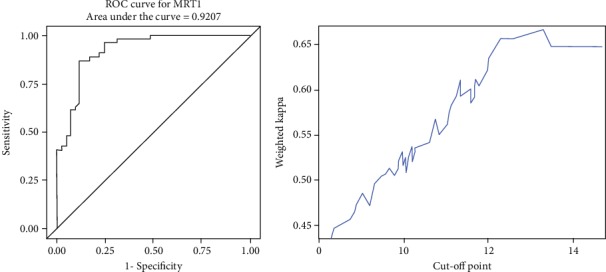
ROC analysis and cut-off value of MRI1.

**Figure 5:**
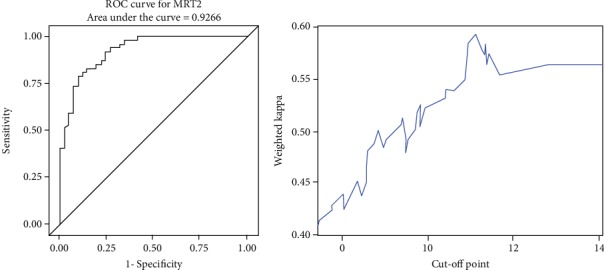
ROC analysis and cut-off value of MRI2.

**Figure 6:**
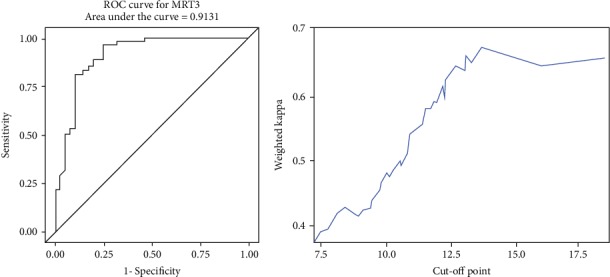
ROC analysis and cut-off value of MRI3.

ROC-curve analysis for each measurement technique was then performed (Figures 4–6). The maximum Youden index (YI) (0.7484) for MRI1 (in terms of discrimination lower/mid third) was given at 7.73 cm corresponding to a sensitivity of 87% and specificity of 87.8%. The maximum weighted Cohen's Kappa for the discrimination of the mid/upper third was given at a cut-off of 13.3 cm.

Maximum YI (0.6985) for MRI2 was given at 7.35 cm corresponding to a sensitivity of 79.63% and specificity of 90.24%. The maximum weighted Cohen's Kappa with defined cut-off for low/mid part of 7.35 cm was detected at 11.15 cm.

Maximum YI (0.7191) for MRI3 was given at 7.11 cm corresponding to a sensitivity of 96.3% and specificity of 75.6%. The maximum weighted Cohen's Kappa with defined cut-off for low/mid part of 7.1 cm was detected at 13.7 cm.

In all, the evaluated cut-off values for MRI were as follows: MRI1 (0-7.7 cm, 7.7-13.3 cm, and >13.3 cm), MRI2 (0-7.4 cm, 7.4-11.2 cm, and >11.2 cm), MRI3 (0-7.1 cm, 7.1-13.7 cm, and >13.7 cm).

#### 3.1.2. Intertechnique Agreement

Analysis of agreement with the rectoscopic classification revealed a weighted Kappa coefficient of 0.66 (95%CL: 0.54-0.78) for MRI1, of 0.59 (95%CL: 0.47-0.72) for MRI2, and of 0.67 (95%CL: 0.55-0.79) for MRI3.

For MRI1 and MRI3, agreement with rigid rectoscopy was substantial (*r* = 0.66 and *r* = 0.67, respectively), whereas MRI2 showed only a moderate agreement (*r* = 0.59). Tables 2, 3, and 4 summarize the results.

## 4. Discussion

Tumor height of rectal cancer is essential for treatment planning and risk stratification. In particular, low rectal cancer is associated with a higher local recurrence rate, higher frequency of positive resection margins, and inferior overall survival [14].

Although MRI is the recommended modality of choice for local tumor staging in rectal cancer, due to its diagnostic accuracy in the assessment of the CRM, only few data report on the accuracy of this technique for measuring the tumor height [8]. The reasons therefore are various: (i) there is, so far, no standardized measurement technique for MRI (curved vs. linear), (ii) the anorectal junction as the recommended baseline reference level by ESMO [3] is not readily identifiable on MRI and thus prone to great variability, and (iii) standard clinical communication refers to the anal verge (AV) and less to the anorectal junction.

Thus, until today, rigid rectoscopy is the method of choice for the assessment of the tumor height in rectal cancer [8]. However, rigid rectoscopy is an uncomfortable and invasive examination for the patient. If it would be possible to assess the tumor height accurately by MRI, rigid rectoscopy could be avoided and MR could serve as a one-stop-shop modality for risk stratification evaluating both CRM and tumor height.

The data of our study indicate that there is only a fair correlation between the gold standard, rigid rectoscopy, and all 3 MRI measurement techniques utilizing the anal verge as the baseline reference level. Since surgeons are generally reporting the distance from the anal verge (AV) to the lower border of the tumor, using the anal verge also in MRI as the baseline reference would ease daily clinical communication. Moreover, the anal verge could be also more steadily identified on MRI than the anorectal junction. Comparing rectoscopy and MRI measurements, Keller et al. reported similar findings to the current study. The authors showed significant difference between rigid rectoscopy and expert MRI measurement of tumor level accordingly [8].

So far, the ESMO defines cut-off values for tumor height as measured by MRI starting at the anorectal junction: low part 0-4 cm, mid part 4-8 cm, and high part > 8 cm [3]. Our study results point to a fair agreement only on an individual basis between the standard UICC rectoscopy measurements of tumor height and either of the used MRI measurement techniques. Thus, utilizing the anal verge as the baseline reference level, we aimed to introduce new cut-off values for defining low, mid, and high rectal cancers for each MRI measurement technique. By doing so, we were able to show a moderate to even substantial agreement between MRI and rigid proctoscopy. Both MRI1, the sum of two unbowed lines, and MRI3, a curved line beginning at the anorectal junction following the anatomical way of the rectum wall, showed substantial agreement with the gold standard method. Therefore, considering the aspect of the ease of use of MRI1 versus MRI3, we propose to use MRI1 as the preferable measuring method for locating the tumor height in daily routine.

Our data indicate that the measurement of the tumor height depends significantly upon the actual position of the patient. Whereas rectoscopy is mostly performed in the lithotomy position or the lateral position with flexed hip, MRI data are acquired in supine with stretched legs. Depending upon the flexion or extension of the hip joint and lower lumbar spine, the rectum is stretched or inflected, which may also affect the different measurement techniques.

There are several limitations of this study. First, the study is retrospective in design. However, since the readers were blinded, it is not to be expected that MRI measurements would have been diverse in a prospective design. Second, the findings presented therein need to be validated by a large multicenter trial. Third, interreader variability between different readers had not been evaluated. Since three different techniques had been evaluated by a nonexpert and correlated by the gold standard method, the data prove clinical usefulness for a broad applicability of the techniques presented therein.

In conclusion, the data of this study illustrate that MRI can be used as a valid method for determining tumor height. Due to the ease of use, we recommend using MRI1, which reveals a substantial correlation to the gold standard, as a one-stop-shop measuring technique for the preoperative evaluation of rectal cancer.

## Figures and Tables

**Table 1:** Mean values ± standard deviations and median min/max for the four different measurement techniques.

	Rigid rectoscopy	*MRI1*	*MRI2*	*MRI3*
*n*	106	107	107	107
Mean (cm)	7.90	8.30	7.42	9.00
SD (cm)	3.499	3.068	2.707	3.967
Median (cm)	8.00	9.02	7.58	9.71
Min (cm)	0	0	0	0
Max (cm)	15.00	14.68	14.05	21.54

**Table 2:** Observed percent agreement: MRI1 (36 + 46 + 2)/106 = 79.25%.

		MRI1			Total
		Low part (0, 7.7)	Mid part (7.7, 13.3)	High part (>13.3)	
Rigid rectoscopy	Low part	36	5	0	**41**
	Mid part	7	46	1	**54**
	High part	0	9	2	**11**

Total		**43**	**60**	**3**	**106**

**Table 3:** Observed percent agreement: MRI2 (37 + 38 + 4)/106 = 74.53%.

		MRI2			Total
		Low part (0, 7.4)	Mid part (7.4, 11.2)	High part (>11.2)	
Rigid rectoscopy	Low part	37	4	0	**41**
	Mid part	13	38	3	**54**
	High part	1	6	4	11

Total		**51**	**48**	**7**	**106**

**Table 4:** Observed percent agreement: MRI3 (31 + 50 + 4)/106 = 80.19%.

		MRI3			Total
		Low part (0, 7.1)	Mid part (7.1, 13.7)	High part (**>**13.7)	
Rigid rectoscopy	Low part	31	10	0	**41**
	Mid part	2	50	2	**54**
	High part	0	7	4	**11**

Total		**33**	**67**	**6**	**106**

## Data Availability

The data used to support the findings of this study are available from the corresponding author upon request.
